# Predicting the Potential Distribution of a Rodent Pest, Brown Rat ( *Rattus norvegicus* ), Associated With Changes in Climate and Land Cover in South Korea

**DOI:** 10.1002/ece3.70573

**Published:** 2024-11-20

**Authors:** Binod Kunwar, Suraj Baral, Young‐Hun Jeong, Seon‐Mi Park, Sung‐Hwan Choi, Hong‐Shik Oh

**Affiliations:** ^1^ Interdisciplinary Graduate Program in Advanced Convergence Technology and Science Jeju National University Jeju Jeju Special Self‐Governing Province Republic of Korea; ^2^ Leibniz Institute for the Analysis of Biodiversity Change Museum Koenig Bonn Bonn Germany; ^3^ Faculty of Science Education Jeju National University Jeju Jeju Special Self‐Governing Province Republic of Korea; ^4^ Research Institute for Basic Science of Jeju National University Jeju National University Jeju Jeju Special Self‐Governing Province Republic of Korea

**Keywords:** climate change, conservation, dispersal scenarios, eco‐geographical variables, MaxEnt, *Rattus norvegicus*

## Abstract

The distribution of mammalian pests is altered by changes in global climate and land cover. *Rattus norvegicus* is a significant pest that contributes to the catastrophic decline of native species. Therefore, the studies identifying potentially suitable habitats for *Rattus norvegicus* and the impact of future climate change on the extent of such habitats are crucial. In this study, we determined the effects of key environmental and ecological variables on *Rattus norvegicus* in South Korea by considering multiple climate changes, land cover, and dispersal scenarios. The available presence locations with the least correlated variables and Maximum Entropy (MaxEnt) model along with multiple Shared Socioeconomic Pathways (SSPs) scenarios were utilized to project current and future habitat suitability. Additionally, three dispersal scenarios were incorporated into the model to enrich the analysis of potential future distribution. Mean diurnal temperature, elevation, and nighttime light were the three most important variables contributing to the species' distribution. The coastal and northern regions of South Korea constitute currently suitable habitats and are expected to exhibit a significant increase in the species' population under future climate projections. The results demonstrate the potential expansion of *Rattus norvegicus* as a result of changes in climate and land cover and provide crucial insights into the species' environmental niches. This study highlights the potential areas for monitoring, early warning, and developing effective prevention and control strategies for *Rattus norvegicus.*

## Graphical abstract

*Rattus norvegicus* is a significant pest that contributes to the catastrophic decline of native species in South Korea. We projected current and future potentially suitable habitats for *R. norvegicus* in South Korea by considering multiple climate changes, land cover, and dispersal scenarios.

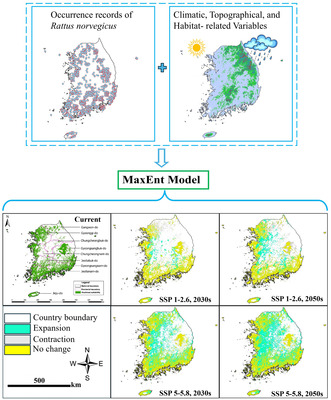

## 1 Introduction

Changes in global climate and land use have significant implications for terrestrial biodiversity, with profound future impacts expected worldwide (Newbold 2018). According to the Sixth Assessment of the Intergovernmental Panel on Climate Change (IPCC), the average global surface temperature is projected to rise by 1.5°C in the near term (2021–2040), with estimates indicating potential warming of up to 4.4°C under high greenhouse gas (GHG) emissions scenarios by the end of the 21st century (Lee et al. 2023). The ecosystem of South Korea is highly vulnerable to climate change, with a projected increase in the mean annual temperature of 0.4°C per decade (Choi et al. 2018). Such a decadal trend would have far‐reaching consequences for rodent expansion, which would negatively impact ecosystems, public health, and food security (Brugueras et al. 2020; Gholamrezaei et al. 2016; Elith, Kearney, and Phillips 2010; Burns, Johnston, and Schmitz 2003). Consequently, it is crucial to understand the projected effects of climate change on rodent pest distributions to facilitate effective risk assessment and management interventions in this regard and implementation (Jacob 2021; Burns, Johnston, and Schmitz 2003). Proactive coping measures to mitigate the adverse impacts of pests, thus safeguarding environmental and human health.

The brown rat (BR; *Rattus norvegicus* ; Mammalia; Muridae) is a typical rodent pest that is widely distributed throughout the globe and, thrives in a variety of urban and rural environments (King, Foster, and Miller 2011; Wilson and ReedeR 2005). The studies on resource selection by BR have suggested an association with wetland habitats, including lakes, ponds, rivers, and streams. Additionally, BR is often found in dense grasslands, scrub, damp forests, agricultural landscapes, and human surroundings (Traweger and Slotta‐Bachmayr 2005; Harper, Dickinson, and Seddon 2005). BR is a notorious pest that cause crop loss and serve as natural hosts for many zoonotic pathogens (Brown, Htwe, and Mulungu 2017; Bonnefoy, Kampen, and Sweeney 2008; Singleton 2003). It is associated with the transmission of *Seoul orthohanatavirus* (hemorrhagic fever with renal syndrome) (Milholland et al. 2018; Kang et al. 2021), Hepatitis E virus (inflammation with hepatic failure) (Park et al. 2024), and *Yersinia pestis* (plague) (Mccormick 2003). It inhabits the entire Korean Peninsula, predominantly coastal areas, agricultural landscapes, and forests (Kim et al. 2013; Jo, Baccus, and Koprowski 2018). BR infestations cause catastrophic declines in the populations of native species worldwide (Harris 2009; Sanders and Maloney 2002; King, Foster, and Miller 2011; Banks and Hughes 2012; Jones et al. 2008; Saunier et al. 2024). In South Korea, several marine breeding birds including the Chinese crested tern (*Thalasseus bernsteini*), a globally critically endangered species, have been disrupted by BR infestations on many uninhabited islands (Gang et al. 2008; Park et al. 2023; Kang et al. 2022). Ensuring the accuracy and reliability of methods used to identify the presence, detection, and management of BR infestations are of utmost importance for the conservation of native species.

Species distribution models (SDMs) are empirical tools used to predict the spatial distribution of species in response to environmental variables (Guillera‐Arroita et al. 2015). These models implement statistical or machine learning algorithms for species occurrence coupled with relevant environmental data to simulate distribution of suitable habitat over space and time (Elith and Leathwick 2009). Various tools and packages are available for SDM application. The maximum entropy model (MaxEnt) is a high‐performance algorithm that is used to determine the potential distribution of a species (Elith et al. 2011). This model is based on the Maximum Entropy theory, which can predict habitat suitability while utilizing occurrence‐only species data only (Phillips, Anderson, and Schapire 2006; Pearson 2006). The robust outcomes and accurate assessments of the method across species have been proven on a global scale. Consequently, MaxEnt has been widely used to predict the potential distributions of rodent pests and perform associated risk assessments (Mohammadi et al. 2019; Bennett and Richard 2021; Hamidi, Mohammadi, and Eskandarzadeh 2018; Lin et al. 2024). Many SDMs assume that species can colonize any suitable habitat irrespective of their original range, and unlimited dispersal (Pearson 2006; Liao et al. 2020). This assumption results in the under‐ or overestimation of species distributions (Engler and Guisan 2009). The consequent estimations of habitat suitability could vary significantly. The dispersal capability of an individual is a crucial factor in simulating a population's potential distribution because of climate change. To address this, we used the MigClim model, which enables the implementation of dispersal constraints in the SDM associated with climate parameters (Engler, Hordijk, and Guisan 2012).

The aim of this study was to assess the impact of climate change on the current and potential future distribution of BR for two benchmark dates (“2030s” and “2050s”, as defined later), based on two Shared Socioeconomic Pathway (SSP) scenarios, namely SSP 1–2.6 and SSP 5–8.5 of the Coupled Model Intercomparison Project Phase 6 (CMIP6) of the IPCC, using MaxEnt applied in South Korea. To the best of our knowledge, a detailed distribution of BR considering bioclimatic, topographic, and habitat‐related variables has not been conducted previously. Thus, our main objectives were (1) to identify current habitat suitability, (2) to identify key environmental and ecological variables that determine BR distribution ranges, (3) to forecast the future distribution as a result of climate change for various dispersal scenarios, and (4) to predict the centroid shift in the potential future distribution. The inclusion of dispersal scenarios in modeling provides a more realistic simulation of the potential distribution in response to environmental and ecological factors. The potential risk areas for BR infestation have been identified, and these projections are crucial for the development and initiation of pest management and public health monitoring policies and strategies in South Korea.

## 2 Materials and Methods

### 2.1 Study Area

**FIGURE 1:**
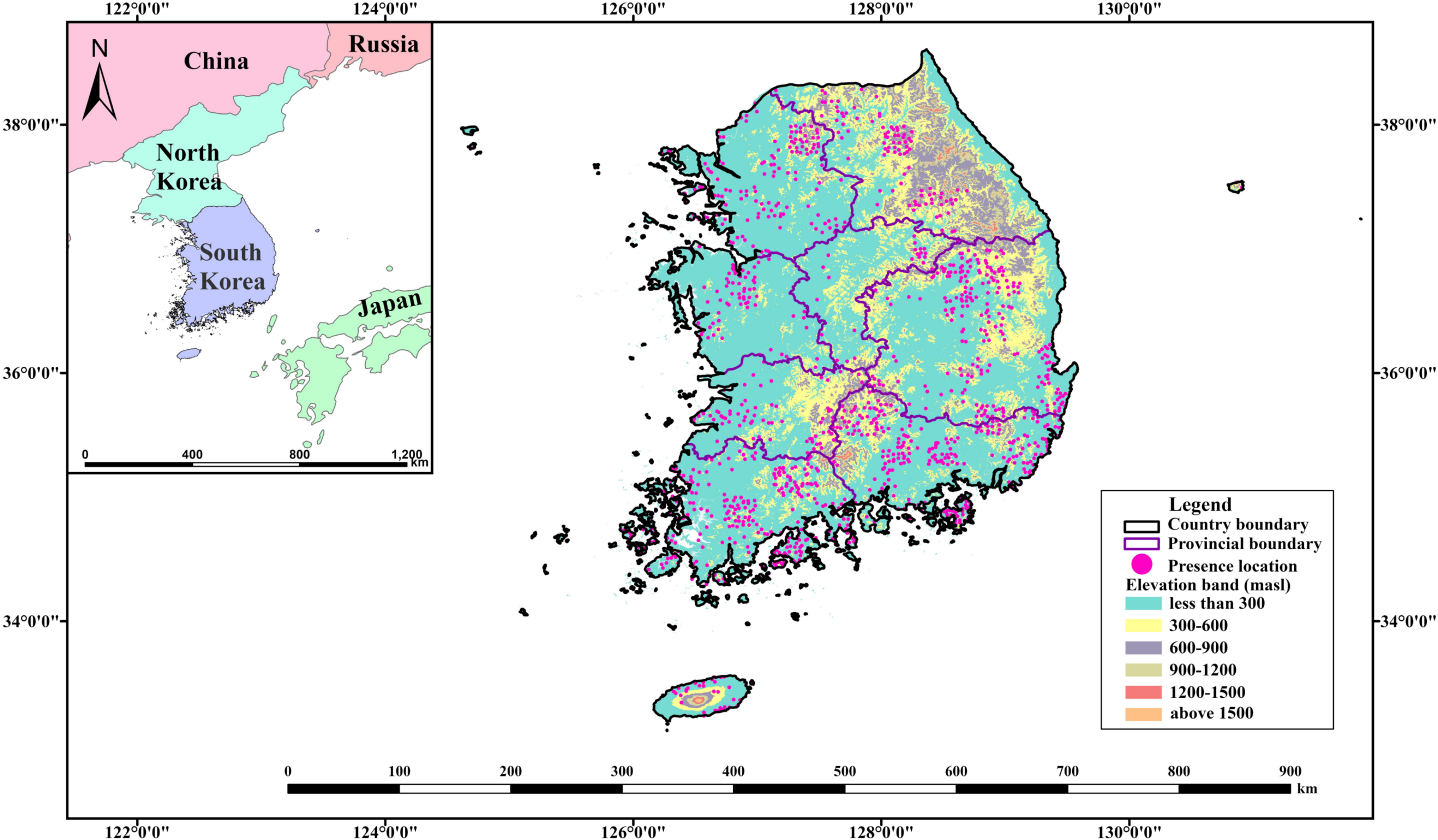
Elevation bands, provincial boundaries, and presence points for MaxEnt model for brown rat (BR) in South Korea, and global location of South Korea (inset).

This study was conducted in South Korea, located in the central part of northeast Asia, adjacent to the Sea of Japan to the east, the Yellow Sea to the west, and the East China Sea to the south (Figure 1). South Korea has a temperate climate with both continental and marine characteristics. The country is mostly mountainous in the northern and eastern parts and low and flat in the southern and western parts. The annual precipitation ranges from 1200 to 1500 mm and is concentrated in the summer. The annual temperature fluctuates significantly, from −6°C to 3°C in the coldest month and 23°C to 26°C in the hottest month. South Korea is divided into three regions (northern, central, and southern) based on the warmth index (Kim, Lee, and Kim 2020). South Korea is rich in biodiversity and hosts 127 mammalian species, including 21 rodent species (Jo, Baccus, and Koprowski 2018).

### 2.2 Species Occurrence and Environmental Variables

Species occurrence data were obtained from the Korean National Mammal Survey (Research 2013) and field‐work resulting in 1346 georeferenced points. Only one occurrence point was retained within each 1 × 1 km grid to avoid clustering effects. We used the spatially rarefy occurrence data tool in SDMtoolbox Pro 0.9.1 (Boria et al. 2014). In total, 415 presence points were acquired for the final projections (Figure 1 and Figure S1 and Table S1).

Environmental variables relating to climate, topography, and habitat influence the species distribution (Taylor et al. 2016). Current and future 19 bioclimatic variables were downloaded from Worldclim version 2.1 (Fick and Hijmans 2017). Shuttle Radar Topography Mission (SRTM)‐derived elevation data of the same resolution were downloaded for the topographic variables (Fick and Hijmans 2017). The slope and aspects were calculated using the downloaded elevation. Moderate Resolution Imaging Spectroradiometer (MODIS) annual landuse and land cover (LULC) data were downloaded from the Google Earth Engine (GEE) (Gray, Sulla‐Menashe, and Friedl 2019), and barren, crop, grassland, forest, and water areas were extracted to create raster layers of the Euclidean distance from the nearest pixels. Annual Normalized Difference Vegetation Index (NDVI) data were downloaded from GEE (Gray, Sulla‐Menashe, and Friedl 2019). Net primary productivity was downloaded from MODIS data (De Leeuw et al. 2019). Nightlight data (https://www.resdc.cn) and global footprint data (WCS and CIESIN 2005) (https://sedac.ciesin.columbia.edu) were included as anthropogenic variables. Coarse fragments (volumetric depth 0–10 cm) (Hengel 2018) was retrieved as soil‐related variables. Future LULC A1B and A2 were downloaded (https://www.resdc.cn) and coupled with low and high GHG emissions scenario respectively (Li et al. 2017; Baral et al. 2023). When predicted data were unavailable, the variables were held constant across the scenarios. All variable data were downloaded at 30 arc‐sec spatial resolutions, similar to those of bioclimatic variables. The spatial analysis function of ArcGIS Pro 3.2.0 was used to extract all the variables for the modeling.

### 2.3 Modeling of Habitat Suitability

A Spearman rank correlation test was performed to address multicollinearity. Highly correlated variables (r ≥ |0.75|) were excluded from the final distribution model (Naimi and Araújo 2016). Among the thirty‐eight variables only nine highly uncorrelated variables were retained for the final projections (Table S2 and Figures S2 and S3). The projection of the species distribution models was conducted by using current climatic variables together with the climatic scenarios under GHG emissions associated with SSP 1–2.6 and 5–8.5 representing low and high emissions scenarios respectively. Two time series averaged for the years: 2030s (2021–2040) and 2050s (2041–2060) were applied for the modeling.

To predict habitat suitability for BR, Maximum Entropy (MaxEnt) version 3.4.0 was used. MaxEnt is one of the best methods available SDM for presence‐only data (Mateo et al. 2010; Merow, Smith, and Silander Jr 2013) and exhibits superior predictability for non‐linear relationships between response and predictor variables (Naimi and Araújo 2016; Phillips, Anderson, and Schapire 2006; Anderson et al. 2006). The model was implemented with 70% of the available presence points, 2500 randomly selected background points, and 10 replicates. The remaining 30% of the available presence points were used for model validation. Two metrics, the area of the curve (AUC) for receiving operating curve value (ROC), and the true skill statistics (TSS) were used to validate the model (Allouche, Tsoar, and Kadmon 2006; Fielding and Bell 1997). The AUC is a threshold‐independent metric which enables the model to distinguish between random and background points (Lawson et al. 2014; Austin 2007; Jiménez‐Valverde, Lobo, and Hortal 2008). The TSS is based on probability threshold which measures the classification performance (Allouche, Tsoar, and Kadmon 2006). We used the “sdm” package in R Studio software for projecting species distribution models under current and future scenarios (Naimi and Araújo 2016). The importance of each variable was established by calculating Pearson's correlation coefficient between predicted and permuted values in the “sdm” package (Naimi and Araújo 2016). Permutation affects the prediction: the higher the importance of a variable, the lower will be the correlation. The relative variable importance was measured using pooled variable importance of all models (Thuiller et al. 2009).

### 2.4 Dispersal Simulation

Integrating dispersal scenarios into SDMs involves incorporating assumption regarding movement patterns across a species' environment. We utilized the R package “MigClim” to determine dispersal limits in the simulation of potential distributions under climate change (Engler, Hordijk, and Guisan 2012). This package implements dispersal constraints in the simulation of species distribution under specific climatic and landscape change scenarios (Engler et al. 2009). The package does not generate the data by itself but is highly compatible with models of habitat suitability (Engler, Hordijk, and Guisan 2012). In our dispersal projection, we used the parameters of the function “MigClim.migrate” (Table 1). The functional parameters included the current map of habitat suitability, map of future habitat suitability, envChgsteps, dispkernel, iniMatAge, PropaguleProd, rcThreshold, and replicateNb. The remaining parameters were set to default values. Each model was replicated 10 times. All predictions were averaged for the final analysis. Following (Baral et al. 2023), we projected the dispersal as low (1 km), medium (5 km) and high (10 km) dispersal scenarios. The low dispersal scenario is equivalent to the minimum known dispersal distance of the species (Hartley and Bishop 1979; Villafañe, Muschetto, and Busch 2008; Heiberg, Sluydts, and Leirs 2012), medium dispersal scenario represents the maximum known distance traveled by BR (Taylor and Quy 1978) and high dispersal scenario reflects an optimistic potential for extended movement (Gardner‐Santana et al. 2009).

**TABLE 1:** Parameter setting for the MigClim model for low dispersal scenario (1 km).

Parameters	Explanation	Parameter Settings
envChgSteps	The number of environmental change steps to perform	2
dispSteps	The number of dispersal steps to perform within each environmental change step	20
dispkernel	The probability of an occupied cell dispersing as a function of distance	1
iniMatAge	The initial maturity age of newly colonized cells	1
PropaguleProd	The probability of an occupied cell producing offspring as a function of time	c (0.1, 0.5, 0.9)
rcThreshold	The threshold value above which a cell is considered suitable	570
replicateNb	The number of times a simulation should be replicated	10

### 2.5 Centroid Shift

The shifting centroid of the potential distribution serves as a crucial marker of a population of organisms' innate adaptability to climate change and their anticipation thereof. The SDM toolbox pro was used to perform a centroid shift in ArcGIS Pro 3.2.0 to calculate centroids of current and potential future distribution (Brown 2014). Through the application of this software, vector files were created that encompassed the magnitude and direction of temporal changes (Brown and Yoder 2015). Finally, we investigated the spatiotemporal changes in BR populations in South Korea by examining the centroid shifts.

## 3 Results

### 3.1 Model Performance and Influencing Variables

We used the MaxEnt model, utilizing 415 georeferenced points (Figure 1) with the nine highly uncorrelated variables to predict the potential and future distributions of BR in South Korea. The ten‐fold cross‐validation yielded a satisfactory AUC score of 0.75 ± 0.05 and TSS score of 0.39 ± 0.02, indicating a reasonably effective performance of the habitat suitability model with no signification deviation in the predictions.

Among the nine variables incorporated in the MaxEnt model, mean diurnal temperature (relative importance = 17.9%), elevation (15.8%), nighttime light (14.9%), normalized difference vegetation index (14.5%), precipitation of the wettest quarter (10.8%), and annual mean temperature (10.2%) constituted the six variables that were strong predictors of distribution and habitat suitability of BR (Table 2). The cumulative contribution of these predictors was 84.1%. Annual precipitation, proportion of the deciduous forest and precipitation of coldest quarter were less important predictors of the distribution (Table 2).

**TABLE 2:** Relative importance of the predictor variables as predicted by the MaxEnt Model for brown rat (BR) presence in South Korea.

Variable	Relative Importance
Climatic	
Bio02 (Mean diurnal temperature)	17.9
Bio16 (Precipitation of wettest quarter)	10.8
Bio01 (Annual mean temperature)	10.2
Bio12 (Annual precipitation)	6.0
Bio19 (Precipitation of coldest quarter)	5.8
Topographical	
Elev (Elevation)	15.8
Habitat‐related	
Nightlight (Nighttime light)	14.9
NDVI (Normalized difference vegetation index)	14.5
Prop_deci (Proportion of deciduous forest)	5.9

### 3.2 Species Response Curves

**FIGURE 2:**
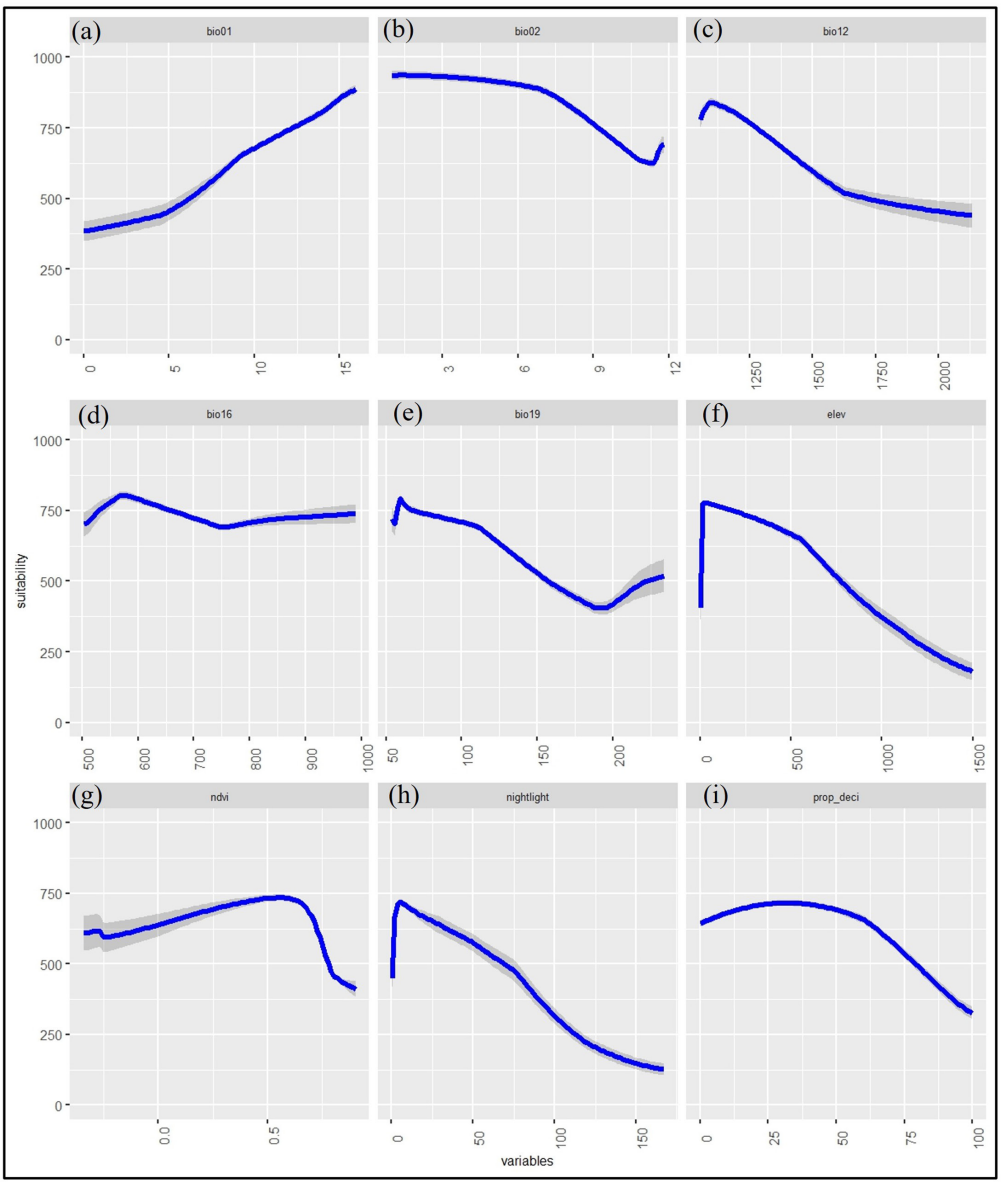
Response curves for the analyzed variables: (a) bio01: Annual mean temperature, (b) Bio02: Mean diurnal range, (c) Bio12: Annual precipitation, (d) Bio16: Precipitation of wettest Quarter, (e) Bio19: Precipitation of coldest quarter, (f) elev: Elevation, (g) ndvi: Normalized difference vegetation index, (h) nightlight: Nighttime Light, and (i) prop_deci: Proportion of deciduous forest. These curves project the distribution of brown rat (BR) using the Maximum Entropy (MaXEnT) model in South Korea.

Based on the response curve of the species, BR exhibited a preference for optimal temperatures ranging from 10°C to 16°C, with a pronounced suitability observed in the 12°C to16°C for annual mean temperature (Figure 2). BR preferred a higher annual diurnal temperature than annual mean temperature. The habitat suitability for BR varied gradually with annual precipitation, peaking between 1625 and 2125 mm. Additionally, BR exhibited a preference for precipitation of the wettest quarter exceeding 900 mm, whereas they favored precipitation of the coldest quarter of less than 100 mm. The BR preferred elevation below 500 m above sea level (asl). The response curve for nighttime light indicated a strong preference for nightlight conditions between 10 and 50 nanowatt/sr/cm^2^ (Figure 2). Furthermore, BR preferred NDVI values ranging from 0.3 to 0.6, and the suitability across the proportion of deciduous forest reached its highest suitability between 25% and 50% (Figure 2).

### 3.3 Species Distribution Modeling

**FIGURE 3:**
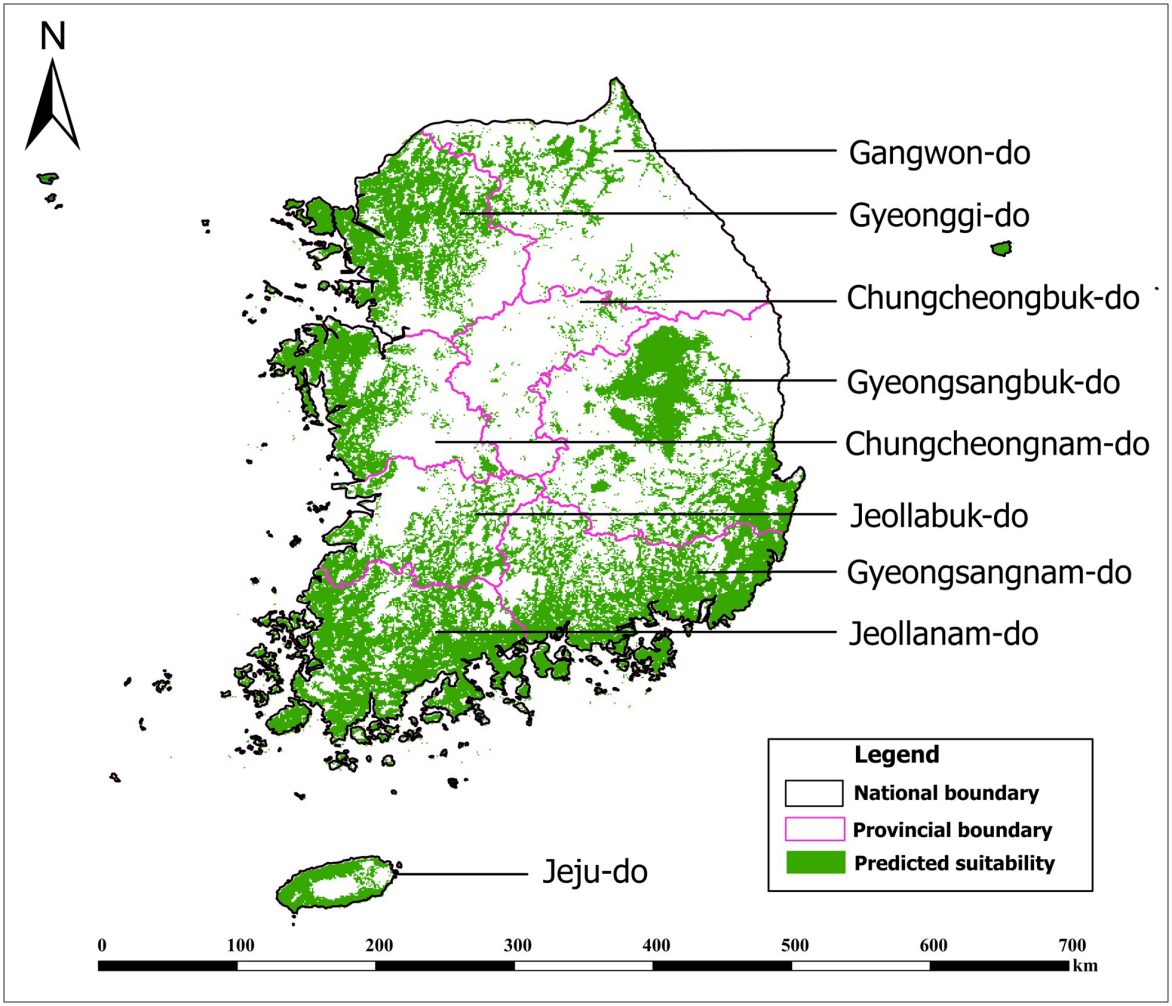
Current distribution of brown rat (BR) in South Korea predicted by the MaxEnt Model after the application of the Maximum True Skill Statistic threshold for presence.

The potential distribution of BR was predicted to be concentrated in the southern and western coastal parts of the mainland and the islands of South Korea. The suitable habitat for BR covered 41.03% of the area of South Korea (Table 3) under the current climatic conditions. The coastal area and some parts of the middle‐southeast regions were found to be suitable for the BR. In particular, areas near large coastline‐based residential areas with grasslands and deciduous forests displayed large suitable areas (Figure 3).

**TABLE 3:** Suitable area for brown rat (BR) in South Korea predicted by MaxEnt model for all the modeled current and future changes, including dispersal scenarios. The area is rounded off to the nearest tenth.

Scenario	Year	Area under unlimited dispersal (km^2^)	Area under modeled dispersal scenarios (km^2^)		
			1 km	5 km	10 km
Current	2024	41,166			
SSP 1–2.6	2030s	36,452	36,299	36,404	36,430
	2050s	44,890	44,047	44,429	44,482
SSP 5–8.5	2030s	62,130	60,521	61,961	61,989
	2050s	71,619	70,535	71,438	71,491

In future climatic scenarios, the habitat suitability of BR was projected to increase across all scenarios, except in low‐emission scenarios for the near future (Table 3). In the low‐emission scenario (SSP1‐2.6) for the 2050s, there is a notable expansion of habitat suitability in the north‐east region consisting of Gyeonggi‐do and Chungcheongnam‐do provinces, and in the northern region consisting of Gyeongsangnam‐do province, whereas a contracting reduction is anticipated in the region for the 2030s (Table 3). For the low‐emission scenarios with unlimited dispersal, approximately 45% of the area of Korea would constitute suitable habitat for BR (Table 3). In the high‐emission scenario (SSP 5–5.8) with unlimited dispersal, the area of suitable habitat for BR would increase significantly, surpassing the current scenario in the near future (Figure 3). In the high‐emissions scenarios, the BR habitat is projected to expand by 35% relative to the current habitat by the 2050s. In the high‐emission scenarios, the area of suitable habitat is expected to occupy an extensive proportion of the mid‐northern regions by the 2050s (Table 3). Overall, 71.4% of the total area of the country would constitute suitable habitat by the 2050s under the high‐emission scenarios.

**FIGURE 4:**
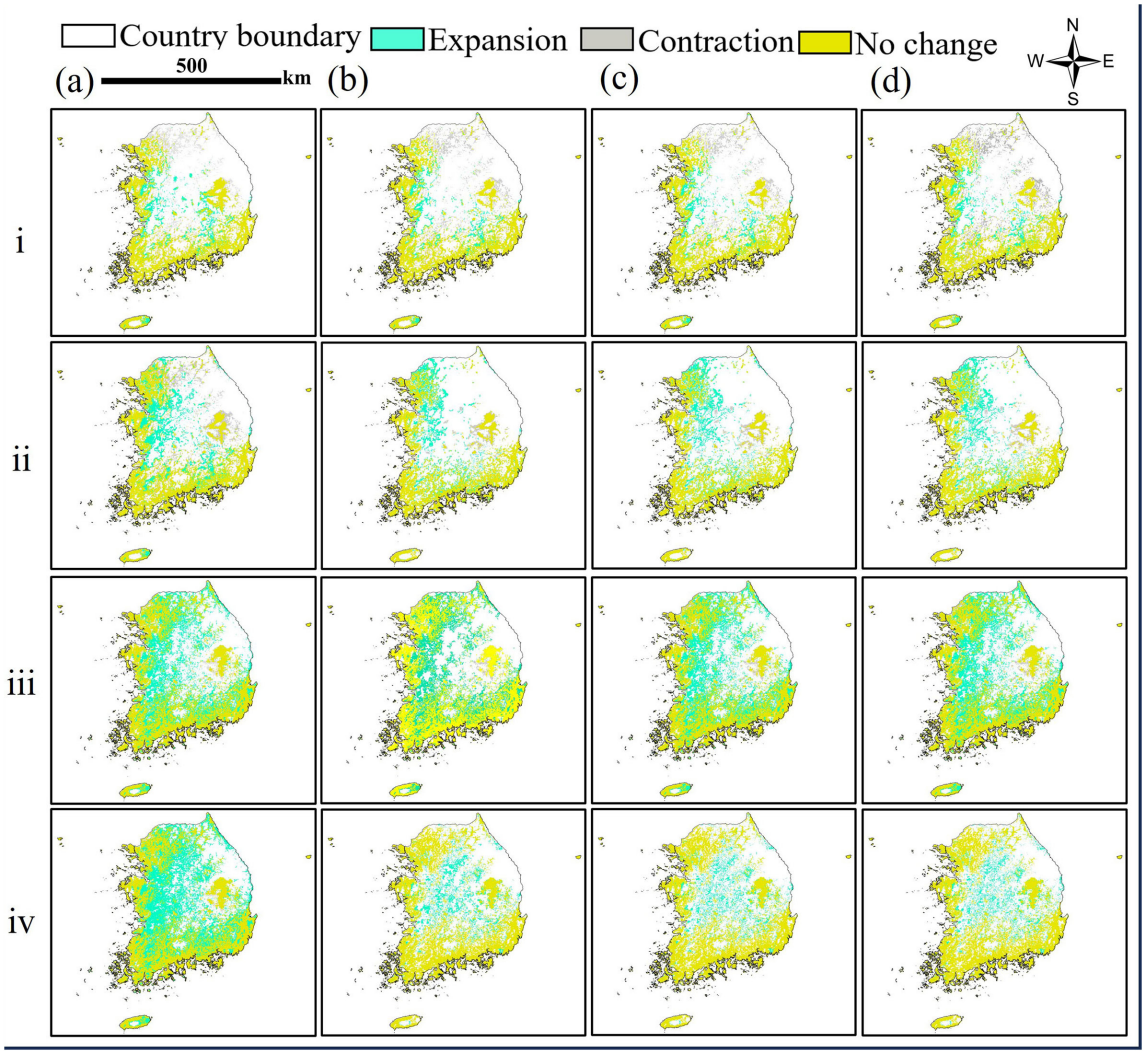
Projection of brown rat (BR) distribution under Shared Socioeconomic Pathways (SSP) (i): SSP 1–2.6, 2030s; (ii): SSP 1–2.6, 2050s; (iii): SSP 5–8.5, 2030s; (iv): SSP 5–8.5, 2050s and dispersal scenarios (a) unlimited, (b) 1 km, (c) 5 km, and (d) 10 km as predicted by MaxEnt Model in South Korea.

The BR habitat displayed a range loss from the current projection for the 2030s under both SSPs 1–2.6 and 5–8.5 under low‐dispersal scenarios, indicating the contraction of suitable habitats in the northern and central regions of South Korea in the future (Table 3). In contrast, when the medium‐dispersal scenario was implemented, there was no significant change in potentially suitable area as compared to the unlimited scenario (Table 3). In addition, in the high‐dispersal scenario, the species could not reach new suitable regions (Table 3, Table S3, and Figure 4). The potential distributions were consistent with the general changes in suitable regions under limited dispersal scenarios (1, 5, and 10 km). Importantly, dispersal distance did not have a significant impact on habitat suitability of BR in South Korea.

### 3.4 Centroid Shift

**FIGURE 5:**
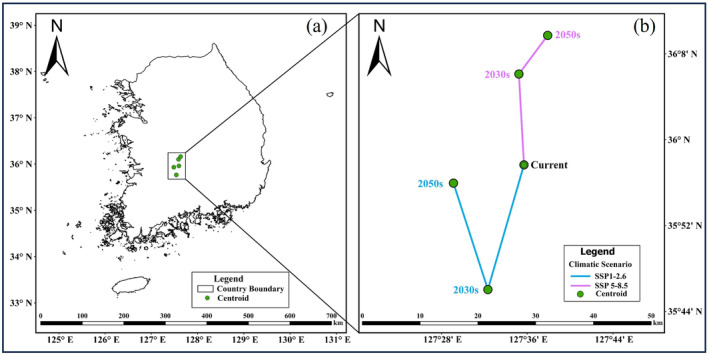
Centroid shift in the potential distribution of brown rat (BR) during the 2030s and 2050s under SPP 1–2.6 and SSP 5–8.5. (a) Centroids, and (b) The green point indicates the centroid of the distribution whereas the line indicates the direction and magnitude of the centroid shift from current to 2030s, and from 2030s to 2050s under SSP 1–2.6 and SSP 5–8.5.

Trajectory changes in the suitable habitat for BR are depicted in Figure 5. Currently, the suitable habitat centroid is located to the north‐east of Jeoksang‐township (127°35′38.4″ E, 35°57′36″ N). Under SSP 1–2.6, there would be a notable shift in the centroid by 22.4 km toward the southern part of Cheoncheon‐myeon by the 2030s. By the 2050s, the centroid would continue moving toward the southern part of Yongdan‐myeon (127°29′6″ E, 35°55′59″ N), by 18.97 km. The shifting rate would be higher by the 2030s at 1.12 km/year (Table S4). Under SSP 5–85, the centroid would shift by 15.5 km toward the south in Jewon‐myeon (127°35′13.2″ E, 35°6′7″ N) for 2030s and a further 7.89 km south‐west in Yangsan‐myeon (127°37′55.2″ E, 36°9′43″ N) by the 2050s. Although the centroid of BR habitat would be projected in various directions under different scenarios, a common trend was a shift toward higher latitudes, except the 2030s scenario.

## 4 Discussion

The main objective of this study was to assess the impact of climate and land cover changes on the potential distribution of BR in South Korea. This study presents the first regional SDM for BR distribution and quantifies the effect of changes in climate and land‐use by incorporating two Shared Socioeconomic Pathways (SSPs 1–2.6 and 5–8.5) scenarios using the MaxEnt model. In addition to the SDMs, we included three dispersal scenarios to provide insights into potential changes in species distribution. The unlimited dispersal model presents an over‐optimistic perspective on species distribution under future climate conditions, whereas, the limited dispersal model yields more accurate projections (Subba et al. 2018). This method has led to a more realistic identification of stable areas than of dynamic areas. These spatially explicit data are crucial for effective conservation strategies and action plans (Subba et al. 2018). Understanding the dynamics of habitat may aid in creating targeted conservation efforts, optimizing resource allocation, and mitigating the adverse effects of climate change on species distribution.

Modeling potential distribution is a valuable tool for assessing habitat suitability and conducting pest risk analyses (Burns, Johnston, and Schmitz 2003). In our study, the MaxEnt model demonstrated a reasonably effective performance, with satisfactory AUC and TSS scores. However, the performance of the model was less effective than that of other rodent models (Lin et al. 2024; Ringani et al. 2022; Wen et al. 2022). This difference in effectiveness might be attributed to the complexity of the model when projecting at large number of localities over time and space (Boria et al. 2014; Lobo, Jiménez‐Valverde, and Real 2008). Additionally, the amount of background data can significantly influence the model's performance of the model (Vanderwal et al. 2009). However, the performance of the model was consistent in the present study, reinforcing the importance of considering the number of localities and the size of the background in developing a robust model.

The future spatial expansion pattern of pest distribution because of climate change deserves attention. Our results showed that the rodent pest BR is predominantly found in urban areas, coastal grasslands, agricultural landscapes, and forests. Future projections under unlimited dispersal scenarios predicted a potential expansion of the BR distribution. Notably, an exception occurred in the low‐emission scenario for the near future, where the results obtained in the present study deviated from the trends observed in other projections. However, such anomalies in future SDM projections are common in regions where precipitation significantly influences distribution patterns. For example, Baral et al. (2023) reported such an anomaly in the distribution extrapolation of yellow monitor ( *Varanus flavescens* ) in Nepal, where precipitation during the wettest quarter was a crucial variable.

South Korea is expected to shift toward a subtropical climate (Kim, Lee, and Kim 2020). The future distribution of BR is expected to increase substantially under different climate change scenarios. A similar case was reported by Lin et al. (2024), who predicted the future distribution of the greater bandicoot rat ( *Bandicota indica* ). In that study, the species was also expected to experience significant habitat gain contributed, primarily driven by annual mean temperature. Comparable findings were reported by Mohammadi et al. (2019) in their projection of the future distribution of two desert jerboas ( *Jaculus blanfordi* and *Jaculus loftusi*) in Iran. The results of those studies are similar to our conclusion (Bennett and Richard 2021; Ringani et al. 2022; Petrosyan et al. 2023). Most of the potential habitat will be occupied under any dispersal scenario indicating that pest problems will continue to be exacerbated in all future scenarios.

MaxEnt identified six key variables influencing the potential distribution of BR in South Korea: three bioclimatic, one topographic, and two habitat‐related variables. The most crucial factors were the diurnal temperature, elevation, and nighttime light. Temperature changes can directly affect the BR population, a factor that tends to dominate urban areas with cold climates, thus supporting our hypothesis (Cavia, Cueto, and Suárez 2009). Cold climates provide suitable growth rates and reproductive conditions for BR (Villarreal, Schlegel, and Prange 2007). Elevation, a key driver of species diversity and distribution patterns, can influence the climatic characteristics of a habitat (Wu et al. 2013). BR preferred habitats below 500 masl, where urban, agricultural landscapes and forested areas are common. Nighttime light ranked third in importance among the variables, highlighting the significance of the habitat‐related variables in BR distribution. Nighttime light provides a unique perspective on urbanization and socioeconomic dynamics, serving as a potential tool for understanding the associated environmental consequences (Zhao et al. 2019). Our prediction indicated an inverse association with nighttime light, similar to the effect of artificial light at night on foraging behavior and vigilance in nocturnal rodents (Zhang et al. 2020). Decrease in annual precipitation and in precipitation during the wettest quarter greatly reduced the probability of BR occurrence, indicating that precipitation is an important constraint on their distribution. BR is likely to occur in wet environments, usually near water sources (Jo, Baccus, and Koprowski 2018; Harper, Dickinson, and Seddon 2005). Furthermore, the intermediate levels of plant productivity (NDVI: 0.3–0.6) and proportion of the deciduous forest preference showed that grasslands and edges of forested areas are suitable for BR (Kim et al. 2013; Jo, Baccus, and Koprowski 2018). Our finding suggested that maintaining higher nightlight intensity without disrupting other organisms, and promoting the growth of healthy dense vegetation can prevent the expansion of BR in South Korea.

We observed that future distribution of BR will shift between 7.89 and 22.40 km. The shifting rates and directions varied among the climatic scenarios, with the fastest shift occurring under SSP 1–2.6 for the 2030s, in an opposite direction compared with other scenarios. Moreover, the final shifts were roughly in the same direction and at higher elevation, as the total suitable area under different scenarios increased compared with the current period. The elevational shift was mainly influenced by temperature and precipitation (Rowe et al. 2015). The response curves showed that both temperature and precipitation significantly contributed for the potential distribution of BR in South Korea. Overall, the temperature indicators were the primary factors for predicting future distribution shifts under climate change. The projection of future range shifts for BR provides valuable insights, enabling relevant departments and decision‐makers to proactively implement preventive measure and effectively monitor the risks of native species extinction, agricultural losses, and zoonotic diseases transmission along these shifting pathways.

The MaxEnt model is highly accurate in determining the current and future status of a species. However, our model was subject to certain uncertainties that should be addressed in future studies. Future bioclimatic variables are likely to vary among scenarios and climatic models, leading to different predictions when a single model is used. Therefore, the use of an ensemble of all possible climatic models is recommended to minimize these discrepancies (Beaumont, Hughes, and Pitman 2008). Our model does not account for complex interactions with other drivers of species dynamics (Bean et al. 2014). Additionally, we adopted a fixed distance as a biologically relevant parameter to determine the potential movement of BR. The MigClim algorithm is based on a cellular automaton and integrates seamlessly with the MaxEnt model (Engler and Guisan 2009). However, the algorithm requires several ecological parameters at the onset of the iterations. Obtaining comprehensive ecological knowledge is often challenging, and can affect model accuracy. Advanced spatially explicit dispersal models, such as agent‐based models, may be appropriate for simulating these behaviors (Baral et al. 2023).

## 5 Conclusion

This study represents the first empirical examination of the ecological niche modeling and expansion risk of BR in South Korea under both current and future climate and landcover change scenarios. Our projections identified a wide range of potentially suitable habitats across the country, including urban regions, coastal grasslands, agricultural landscapes, and forests. The key environmental variables influencing the distribution of BR are annual diurnal temperature, elevation, and nighttime light. Additionally, our findings provide comprehensive insights into the regions where this rodent pest is likely to expand under future climatic scenarios. These results can be used to identify high‐risk areas and to prioritize conservation efforts. Consequently, our findings could assist ecological managers in promptly implementing measures to prevent the future spread of BR in South Korea. This information is also valuable for developing action plans and ecosystem management strategies, as well as guiding future surveys and monitoring activities.

## Author Contributions

**Binod Kunwar:** conceptualization (lead), data curation (lead), formal analysis (lead), funding acquisition (equal), methodology (lead), validation (lead), writing – original draft (lead), writing – review and editing (equal). **Suraj Baral:** conceptualization (lead), formal analysis (equal), funding acquisition (supporting), methodology (equal), validation (equal), writing – original draft (supporting), writing – review and editing (equal). **Young‐Hun Jeong:** data curation (supporting), funding acquisition (equal), methodology (supporting), writing – review and editing (supporting). **Seon‐Mi Park:** data curation (supporting), funding acquisition (equal), methodology (supporting), writing – review and editing (supporting). **Sung‐Hwan Choi:** data curation (supporting), funding acquisition (equal), methodology (supporting), writing – review and editing (supporting). **Hong‐Shik Oh:** conceptualization (equal), data curation (equal), funding acquisition (equal), supervision (lead), writing – original draft (equal).

## Conflicts of Interest

The authors declare no conflicts of interest.

## Supporting information


Appendix S1.

## Data Availability

The data that support this study has been provided in Supporting Informations.
