# Supplementary material for: Predicting the Potential Distribution of a Rodent Pest, Brown Rat ( Rattus norvegicus ), Associated With Changes in Climate and Land Cover in South Korea
Source: Ecol Evol. 2024 Nov 20;14(11):e70573. doi: 10.1002/ece3.70573 (PMC11578634; doi:10.1002/ece3.70573)
Supplement: Supplementary file 1 — Appendix S1. [file ECE3-14-e70573-s001.docx]

**Supplementary Information:**

**Predicting the potential distribution of a rodent pest, brown rat (*Rattus* *norvegicus*), associated with changes in climate and land cover in South Korea**

**Binod Kunwar,^1^ Suraj Baral,^2^ Young-Hun Jeong,^4^** **Seon-Mi Park,^3^** **Sung-Hwan Choi^4^ Hong-Shik Oh^1,4*^**

^1^Interdisciplinary Graduate Program in Advanced Convergence technology and Science, Jeju National University, Jeju Special Self-Governing Province, 63243, Republic of Korea

^2^ Herpetology section, Leibniz Institute for the Analysis of Biodiversity Change, Museum Koenig Bonn, Germany

^3^Research Institute for Basic Science of Jeju National University, Jeju National University, Jeju Special Self-Governing Province, 63243, Republic of Korea

^4^Faculty of Science Education, Jeju National University, Jeju Special Self-Governing Province, 63243, Republic of Korea

Contents:

1. FIGURE S1 Human settlement and presence points of brown rat (BR) in South Korea.2.
2. TABLE S1. Georeferenced Points of *Rattus* *Norvegicus* in South Korea.
3. TABLE S2. Environmental variables used for habitat suitability modelling of brown rat (BR) in South Korea.
4. FIGURE S2 Correlation matrix plot of selected variables for habitat suitability modelling of brown rat (BR). Highly correlated variables (r ≥ |0.75|) were excluded for the final distribution model.
5. FIGURE S3 Spearman’s Rank Correlation between the final set of variables used for the ensemble models for the brown rat (BR). The variables are coded as bio01: Annual Mean Temperature, Bio02: Mean Diurnal Range, Bio12: Annual precipitation, Bio16: Precipitation of wettest Quarter, Bio19: Precipitation of Coldest Quarter, nightlight: Nighttime Light, elev: elevation, ndvi: Normalized Difference Vegetation Index, and prop_deci: Proportion of deciduous forest.
6. TABLE S3. Distribution changes of brown rat (BR) based on binary distribution for 2030s and 2050s under the shared socioeconomic pathways (SSPs) 1-2.6 and 5-8.5 including dispersal scenarios.
7. TABLE S4. centroid coordinates and shift range of brown rat (BR) in the South Korea.


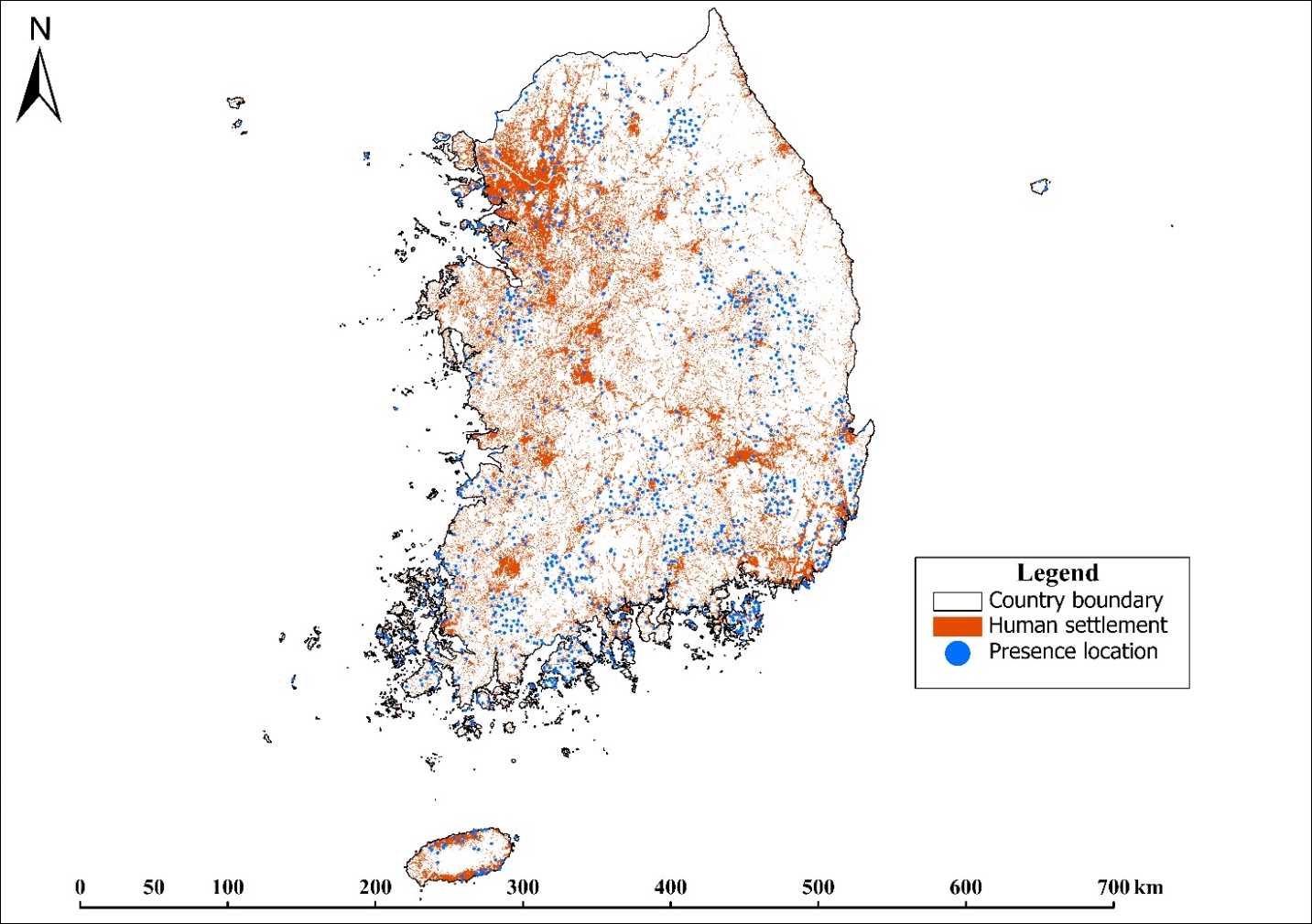


FIGURE S1 Human Settlement and presence points of brown rat (BR) in South Korea

TABLE S1. Georeferenced Points of *Rattus* *norvegicus* in South Korea.

| **S.N.** | **Species** | **Longitude (˚E)** | **Latitude (˚N)** |
| --- | --- | --- | --- |
| 1 | *Rattus norvegicus* | 128.857225 | 36.50419167 |
| 2 | *Rattus norvegicus* | 128.7649417 | 36.61003333 |
| 3 | *Rattus norvegicus* | 128.9352556 | 36.64776389 |
| 4 | *Rattus norvegicus* | 128.8176722 | 36.72217778 |
| 5 | *Rattus norvegicus* | 128.6675139 | 35.26833889 |
| 6 | *Rattus norvegicus* | 128.221775 | 36.61279722 |
| 7 | *Rattus norvegicus* | 129.0518556 | 36.52435 |
| 8 | *Rattus norvegicus* | 129.1065639 | 36.64620556 |
| 9 | *Rattus norvegicus* | 129.0111361 | 36.74410833 |
| 10 | *Rattus norvegicus* | 127.6403944 | 38.14196389 |
| 11 | *Rattus norvegicus* | 127.6912444 | 38.00379167 |
| 12 | *Rattus norvegicus* | 127.5197472 | 38.07153333 |
| 13 | *Rattus norvegicus* | 127.5472861 | 38.25605278 |
| 14 | *Rattus norvegicus* | 129.0884583 | 35.05655556 |
| 15 | *Rattus norvegicus* | 128.5563083 | 35.38972778 |
| 16 | *Rattus norvegicus* | 128.6167861 | 35.47741389 |
| 17 | *Rattus norvegicus* | 129.1883639 | 35.16400278 |
| 18 | *Rattus norvegicus* | 128.535775 | 35.26318333 |
| 19 | *Rattus norvegicus* | 128.3826694 | 35.26633611 |
| 20 | *Rattus norvegicus* | 128.38405 | 35.36585278 |
| 21 | *Rattus norvegicus* | 127.6436944 | 35.25723056 |
| 22 | *Rattus norvegicus* | 127.4814194 | 36.59074444 |
| 23 | *Rattus norvegicus* | 128.2209222 | 35.39385556 |
| 24 | *Rattus norvegicus* | 128.2228389 | 35.28584167 |
| 25 | *Rattus norvegicus* | 128.06255 | 35.28878611 |
| 26 | *Rattus norvegicus* | 129.3444722 | 35.37291944 |
| 27 | *Rattus norvegicus* | 129.417675 | 35.49355 |
| 28 | *Rattus norvegicus* | 129.2242056 | 35.26042222 |
| 29 | *Rattus norvegicus* | 129.0736528 | 35.27616389 |
| 30 | *Rattus norvegicus* | 129.1393694 | 35.39191944 |
| 31 | *Rattus norvegicus* | 129.1769472 | 35.49018611 |
| 32 | *Rattus norvegicus* | 128.8939611 | 35.33752222 |
| 33 | *Rattus norvegicus* | 127.4078944 | 35.26638333 |
| 34 | *Rattus norvegicus* | 127.4557583 | 35.38843889 |
| 35 | *Rattus norvegicus* | 127.4707583 | 35.49169167 |
| 36 | *Rattus norvegicus* | 127.6159806 | 35.46051944 |
| 37 | *Rattus norvegicus* | 127.1490528 | 38.27638333 |
| 38 | *Rattus norvegicus* | 127.2035694 | 38.13259167 |
| 39 | *Rattus norvegicus* | 128.0188389 | 36.74643333 |
| 40 | *Rattus norvegicus* | 127.9534333 | 36.66718333 |
| 41 | *Rattus norvegicus* | 128.0752139 | 36.55305833 |
| 42 | *Rattus norvegicus* | 128.6900361 | 36.73616667 |
| 43 | *Rattus norvegicus* | 128.5838694 | 36.5182 |
| 44 | *Rattus norvegicus* | 126.91125 | 38.02918611 |
| 45 | *Rattus norvegicus* | 126.9957917 | 38.10596944 |
| 46 | *Rattus norvegicus* | 126.7267417 | 37.90108889 |
| 47 | *Rattus norvegicus* | 127.7825278 | 38.13691111 |
| 48 | *Rattus norvegicus* | 127.9856056 | 38.06828611 |
| 49 | *Rattus norvegicus* | 127.8401833 | 38.28446944 |
| 50 | *Rattus norvegicus* | 127.9595778 | 38.22616389 |
| 51 | *Rattus norvegicus* | 126.7161222 | 36.2808 |
| 52 | *Rattus norvegicus* | 126.5377917 | 36.27746389 |
| 53 | *Rattus norvegicus* | 126.6175111 | 36.36746389 |
| 54 | *Rattus norvegicus* | 126.6877861 | 36.48496389 |
| 55 | *Rattus norvegicus* | 126.9661917 | 37.02193611 |
| 56 | *Rattus norvegicus* | 126.8031361 | 37.04876944 |
| 57 | *Rattus norvegicus* | 126.5328611 | 37.01804167 |
| 58 | *Rattus norvegicus* | 126.6868583 | 37.12276667 |
| 59 | *Rattus norvegicus* | 127.7824167 | 36.34308611 |
| 60 | *Rattus norvegicus* | 128.2219694 | 37.65563889 |
| 61 | *Rattus norvegicus* | 128.1602139 | 37.52697222 |
| 62 | *Rattus norvegicus* | 126.920325 | 37.23079444 |
| 63 | *Rattus norvegicus* | 127.4813528 | 35.09253056 |
| 64 | *Rattus norvegicus* | 127.3575694 | 35.00313889 |
| 65 | *Rattus norvegicus* | 127.3010667 | 35.12079167 |
| 66 | *Rattus norvegicus* | 127.2063667 | 35.03399444 |
| 67 | *Rattus norvegicus* | 127.2040806 | 35.22642778 |
| 68 | *Rattus norvegicus* | 127.3969861 | 36.36509167 |
| 69 | *Rattus norvegicus* | 127.5193944 | 36.30761111 |
| 70 | *Rattus norvegicus* | 126.7340889 | 35.02000278 |
| 71 | *Rattus norvegicus* | 126.7953083 | 35.22136389 |
| 72 | *Rattus norvegicus* | 126.4950667 | 36.452825 |
| 73 | *Rattus norvegicus* | 126.7402583 | 34.82133889 |
| 74 | *Rattus norvegicus* | 126.59265 | 34.75839167 |
| 75 | *Rattus norvegicus* | 126.596175 | 34.89066944 |
| 76 | *Rattus norvegicus* | 127.3781139 | 36.49419444 |
| 77 | *Rattus norvegicus* | 126.9945556 | 34.77997222 |
| 78 | *Rattus norvegicus* | 126.9656611 | 34.89791667 |
| 79 | *Rattus norvegicus* | 126.8221583 | 34.90101667 |
| 80 | *Rattus norvegicus* | 126.8686583 | 34.99764167 |
| 81 | *Rattus norvegicus* | 125.9838889 | 36.11683889 |
| 82 | *Rattus norvegicus* | 126.4642833 | 35.16452778 |
| 83 | *Rattus norvegicus* | 126.5069806 | 35.03105556 |
| 84 | *Rattus norvegicus* | 126.6088667 | 35.10172222 |
| 85 | *Rattus norvegicus* | 126.3275056 | 35.14633056 |
| 86 | *Rattus norvegicus* | 126.3158972 | 35.00635833 |
| 87 | *Rattus norvegicus* | 127.9464722 | 35.14413889 |
| 88 | *Rattus norvegicus* | 127.9060972 | 35.05795278 |
| 89 | *Rattus norvegicus* | 126.4005389 | 34.82944444 |
| 90 | *Rattus norvegicus* | 126.604325 | 37.473675 |
| 91 | *Rattus norvegicus* | 126.488325 | 37.27197778 |
| 92 | *Rattus norvegicus* | 126.3714778 | 37.45341389 |
| 93 | *Rattus norvegicus* | 127.6751361 | 37.555275 |
| 94 | *Rattus norvegicus* | 127.7326611 | 37.72790278 |
| 95 | *Rattus norvegicus* | 127.5000944 | 37.53082222 |
| 96 | *Rattus norvegicus* | 127.5213722 | 37.67409722 |
| 97 | *Rattus norvegicus* | 126.9889806 | 37.38575833 |
| 98 | *Rattus norvegicus* | 126.9772667 | 37.49773889 |
| 99 | *Rattus norvegicus* | 126.780175 | 37.46864444 |
| 100 | *Rattus norvegicus* | 126.7394083 | 37.28536389 |
| 101 | *Rattus norvegicus* | 127.2849722 | 34.90033333 |
| 102 | *Rattus norvegicus* | 127.4791111 | 34.95119444 |
| 103 | *Rattus norvegicus* | 127.3889167 | 34.79536111 |
| 104 | *Rattus norvegicus* | 127.6087778 | 34.94808333 |
| 105 | *Rattus norvegicus* | 127.7127778 | 34.88613889 |
| 106 | *Rattus norvegicus* | 127.5693722 | 34.77715278 |
| 107 | *Rattus norvegicus* | 127.7277222 | 34.77447222 |
| 108 | *Rattus norvegicus* | 127.8819444 | 34.84194444 |
| 109 | *Rattus norvegicus* | 127.5588056 | 34.68027778 |
| 110 | *Rattus norvegicus* | 127.7415278 | 34.63441667 |
| 111 | *Rattus norvegicus* | 128.2209444 | 34.98027778 |
| 112 | *Rattus norvegicus* | 128.1368056 | 34.90397222 |
| 113 | *Rattus norvegicus* | 128.2260278 | 34.81852778 |
| 114 | *Rattus norvegicus* | 128.3498889 | 34.95591667 |
| 115 | *Rattus norvegicus* | 128.53335 | 34.96423889 |
| 116 | *Rattus norvegicus* | 128.6872028 | 34.96547778 |
| 117 | *Rattus norvegicus* | 128.5314528 | 34.85705278 |
| 118 | *Rattus norvegicus* | 128.7330833 | 34.84431111 |
| 119 | *Rattus norvegicus* | 128.6069639 | 34.73455556 |
| 120 | *Rattus norvegicus* | 128.1115167 | 35.08756389 |
| 121 | *Rattus norvegicus* | 127.1078861 | 36.18743889 |
| 122 | *Rattus norvegicus* | 127.2353056 | 36.13276389 |
| 123 | *Rattus norvegicus* | 127.0520028 | 36.08884167 |
| 124 | *Rattus norvegicus* | 127.4816806 | 36.11304722 |
| 125 | *Rattus norvegicus* | 128.567375 | 36.42461389 |
| 126 | *Rattus norvegicus* | 128.6624972 | 36.35113889 |
| 127 | *Rattus norvegicus* | 128.6523639 | 36.25653889 |
| 128 | *Rattus norvegicus* | 128.9783944 | 36.44028611 |
| 129 | *Rattus norvegicus* | 128.8024556 | 36.34892222 |
| 130 | *Rattus norvegicus* | 128.9645694 | 36.33635833 |
| 131 | *Rattus norvegicus* | 127.1124722 | 37.309 |
| 132 | *Rattus norvegicus* | 127.4095833 | 37.34363889 |
| 133 | *Rattus norvegicus* | 127.989175 | 37.46764167 |
| 134 | *Rattus norvegicus* | 127.9669972 | 37.35641389 |
| 135 | *Rattus norvegicus* | 128.2269778 | 37.34987778 |
| 136 | *Rattus norvegicus* | 128.3238889 | 37.46921667 |
| 137 | *Rattus norvegicus* | 128.4861056 | 37.44788056 |
| 138 | *Rattus norvegicus* | 128.4077861 | 37.36683056 |
| 139 | *Rattus norvegicus* | 128.4466194 | 37.25674444 |
| 140 | *Rattus norvegicus* | 126.9680556 | 35.98416667 |
| 141 | *Rattus norvegicus* | 126.8980556 | 35.90583333 |
| 142 | *Rattus norvegicus* | 126.9022222 | 35.7775 |
| 143 | *Rattus norvegicus* | 127.1127778 | 35.93527778 |
| 144 | *Rattus norvegicus* | 127.1831111 | 37.17141667 |
| 145 | *Rattus norvegicus* | 127.4371417 | 37.2029 |
| 146 | *Rattus norvegicus* | 126.454275 | 35.82062222 |
| 147 | *Rattus norvegicus* | 126.7325556 | 35.86294167 |
| 148 | *Rattus norvegicus* | 128.1178611 | 36.09672222 |
| 149 | *Rattus norvegicus* | 127.5853528 | 37.16812222 |
| 150 | *Rattus norvegicus* | 127.0374444 | 34.44333333 |
| 151 | *Rattus norvegicus* | 127.1910556 | 34.71147222 |
| 152 | *Rattus norvegicus* | 126.6425833 | 33.54391667 |
| 153 | *Rattus norvegicus* | 126.8081667 | 33.36944444 |
| 154 | *Rattus norvegicus* | 126.6490833 | 33.43513889 |
| 155 | *Rattus norvegicus* | 126.6998889 | 33.30186111 |
| 156 | *Rattus norvegicus* | 127.2868361 | 34.49351944 |
| 157 | *Rattus norvegicus* | 127.1354167 | 34.55342222 |
| 158 | *Rattus norvegicus* | 127.4401861 | 34.52405 |
| 159 | *Rattus norvegicus* | 127.3229444 | 34.68055556 |
| 160 | *Rattus norvegicus* | 129.0981 | 36.15634444 |
| 161 | *Rattus norvegicus* | 126.9220111 | 34.59988056 |
| 162 | *Rattus norvegicus* | 126.7771806 | 34.666225 |
| 163 | *Rattus norvegicus* | 128.7372028 | 36.06694444 |
| 164 | *Rattus norvegicus* | 128.8724611 | 36.06306111 |
| 165 | *Rattus norvegicus* | 129.3875472 | 36.07351389 |
| 166 | *Rattus norvegicus* | 129.322875 | 36.21225 |
| 167 | *Rattus norvegicus* | 128.6008778 | 37.39506944 |
| 168 | *Rattus norvegicus* | 128.6906 | 37.46679167 |
| 169 | *Rattus norvegicus* | 127.7640861 | 36.01016944 |
| 170 | *Rattus norvegicus* | 127.6428861 | 36.10172778 |
| 171 | *Rattus norvegicus* | 127.7853556 | 36.11854167 |
| 172 | *Rattus norvegicus* | 126.3876944 | 33.34508889 |
| 173 | *Rattus norvegicus* | 126.5683806 | 33.23760556 |
| 174 | *Rattus norvegicus* | 126.3651778 | 33.46194444 |
| 175 | *Rattus norvegicus* | 126.9480056 | 33.50938611 |
| 176 | *Rattus norvegicus* | 127.5163528 | 35.96331111 |
| 177 | *Rattus norvegicus* | 128.3703028 | 35.85017222 |
| 178 | *Rattus norvegicus* | 128.0152611 | 35.901 |
| 179 | *Rattus norvegicus* | 126.4723028 | 34.33853611 |
| 180 | *Rattus norvegicus* | 126.6138167 | 34.36921667 |
| 181 | *Rattus norvegicus* | 126.6174528 | 34.20049722 |
| 182 | *Rattus norvegicus* | 126.7283833 | 34.34212778 |
| 183 | *Rattus norvegicus* | 128.5647 | 35.85683889 |
| 184 | *Rattus norvegicus* | 128.5327639 | 35.99719444 |
| 185 | *Rattus norvegicus* | 128.5710972 | 37.13803889 |
| 186 | *Rattus norvegicus* | 126.3216667 | 34.69 |
| 187 | *Rattus norvegicus* | 126.3619444 | 34.56388889 |
| 188 | *Rattus norvegicus* | 126.2333333 | 34.91416667 |
| 189 | *Rattus norvegicus* | 129.2711833 | 35.96806667 |
| 190 | *Rattus norvegicus* | 129.4124528 | 35.83988889 |
| 191 | *Rattus norvegicus* | 125.2846444 | 34.43631944 |
| 192 | *Rattus norvegicus* | 125.900575 | 34.73488333 |
| 193 | *Rattus norvegicus* | 126.0794083 | 34.73928056 |
| 194 | *Rattus norvegicus* | 126.1676694 | 34.63995556 |
| 195 | *Rattus norvegicus* | 126.0628528 | 34.55450278 |
| 196 | *Rattus norvegicus* | 126.2276611 | 34.45868889 |
| 197 | *Rattus norvegicus* | 126.4851333 | 34.46186667 |
| 198 | *Rattus norvegicus* | 126.3352389 | 34.41163333 |
| 199 | *Rattus norvegicus* | 130.8610833 | 37.53516111 |
| 200 | *Rattus norvegicus* | 129.2961694 | 35.77145833 |
| 201 | *Rattus norvegicus* | 128.693125 | 35.92899444 |
| 202 | *Rattus norvegicus* | 128.65925 | 35.76729722 |
| 203 | *Rattus norvegicus* | 127.8960639 | 35.76101944 |
| 204 | *Rattus norvegicus* | 128.2658889 | 37.10291389 |
| 205 | *Rattus norvegicus* | 129.5033611 | 36.02547222 |
| 206 | *Rattus norvegicus* | 126.5250139 | 34.65188611 |
| 207 | *Rattus norvegicus* | 127.3478 | 37.88003611 |
| 208 | *Rattus norvegicus* | 127.4778028 | 37.958925 |
| 209 | *Rattus norvegicus* | 127.310025 | 37.99003889 |
| 210 | *Rattus norvegicus* | 127.348075 | 37.77503611 |
| 211 | *Rattus norvegicus* | 127.7708194 | 35.50709167 |
| 212 | *Rattus norvegicus* | 126.4934194 | 35.63463056 |
| 213 | *Rattus norvegicus* | 127.9373611 | 35.62174722 |
| 214 | *Rattus norvegicus* | 127.6847972 | 35.7319 |
| 215 | *Rattus norvegicus* | 127.5125917 | 35.60191389 |
| 216 | *Rattus norvegicus* | 127.5720472 | 36.94892778 |
| 217 | *Rattus norvegicus* | 127.5306083 | 36.85279722 |
| 218 | *Rattus norvegicus* | 127.1001167 | 36.95175556 |
| 219 | *Rattus norvegicus* | 126.9767944 | 36.85398889 |
| 220 | *Rattus norvegicus* | 126.8618056 | 36.77816389 |
| 221 | *Rattus norvegicus* | 128.0391556 | 35.74537222 |
| 222 | *Rattus norvegicus* | 126.8449611 | 35.670625 |
| 223 | *Rattus norvegicus* | 128.1943972 | 35.67771111 |
| 224 | *Rattus norvegicus* | 126.717775 | 35.59104722 |
| 225 | *Rattus norvegicus* | 127.9425556 | 35.50761944 |
| 226 | *Rattus norvegicus* | 127.4903 | 37.84586667 |
| 227 | *Rattus norvegicus* | 126.6085056 | 35.7232 |
| 228 | *Rattus norvegicus* | 126.9482361 | 35.5967 |
| 229 | *Rattus norvegicus* | 126.4807389 | 35.51261111 |
| 230 | *Rattus norvegicus* | 127.15345 | 35.73188611 |
| 231 | *Rattus norvegicus* | 127.0906333 | 35.59756667 |
| 232 | *Rattus norvegicus* | 127.6436389 | 35.5622 |
| 233 | *Rattus norvegicus* | 127.1426056 | 37.86581667 |
| 234 | *Rattus norvegicus* | 127.6816667 | 37.89426389 |
| 235 | *Rattus norvegicus* | 128.1675333 | 35.54545278 |
| 236 | *Rattus norvegicus* | 127.8259528 | 35.65836944 |
| 237 | *Rattus norvegicus* | 126.8523639 | 36.63679167 |
| 238 | *Rattus norvegicus* | 126.7520944 | 36.70039444 |
| 239 | *Rattus norvegicus* | 126.9779083 | 36.728725 |
| 240 | *Rattus norvegicus* | 126.9840194 | 36.54763611 |
| 241 | *Rattus norvegicus* | 126.5991361 | 36.57190833 |
| 242 | *Rattus norvegicus* | 126.5718778 | 36.69076944 |
| 243 | *Rattus norvegicus* | 126.6012806 | 37.64477222 |
| 244 | *Rattus norvegicus* | 128.9505472 | 35.54189444 |
| 245 | *Rattus norvegicus* | 128.8075194 | 35.53331389 |
| 246 | *Rattus norvegicus* | 128.9833278 | 35.65245278 |
| 247 | *Rattus norvegicus* | 128.809575 | 35.65739722 |
| 248 | *Rattus norvegicus* | 128.2077861 | 37.78028056 |
| 249 | *Rattus norvegicus* | 128.0533556 | 37.78472222 |
| 250 | *Rattus norvegicus* | 128.2230611 | 37.88943333 |
| 251 | *Rattus norvegicus* | 128.126125 | 37.97775556 |
| 252 | *Rattus norvegicus* | 128.0505778 | 37.89943056 |
| 253 | *Rattus norvegicus* | 126.6762778 | 35.44886944 |
| 254 | *Rattus norvegicus* | 126.6323944 | 35.300275 |
| 255 | *Rattus norvegicus* | 126.2297722 | 37.77623889 |
| 256 | *Rattus norvegicus* | 129.2217583 | 35.63246389 |
| 257 | *Rattus norvegicus* | 129.4341083 | 35.62030556 |
| 258 | *Rattus norvegicus* | 129.4369139 | 35.71819444 |
| 259 | *Rattus norvegicus* | 128.6351194 | 36.96243333 |
| 260 | *Rattus norvegicus* | 128.6392583 | 36.84777778 |
| 261 | *Rattus norvegicus* | 128.8900139 | 36.82364444 |
| 262 | *Rattus norvegicus* | 128.8731806 | 36.985625 |
| 263 | *Rattus norvegicus* | 128.7749389 | 36.91271667 |
| 264 | *Rattus norvegicus* | 128.5306306 | 36.77973056 |
| 265 | *Rattus norvegicus* | 128.3219333 | 35.55038056 |
| 266 | *Rattus norvegicus* | 128.3581556 | 35.71418333 |
| 267 | *Rattus norvegicus* | 127.1047694 | 35.46373056 |
| 268 | *Rattus norvegicus* | 126.8975639 | 35.40940278 |
| 269 | *Rattus norvegicus* | 126.976725 | 35.307525 |
| 270 | *Rattus norvegicus* | 126.4465111 | 35.35856111 |
| 271 | *Rattus norvegicus* | 128.3381778 | 36.88811389 |
| 272 | *Rattus norvegicus* | 128.3472917 | 37.00075278 |
| 273 | *Rattus norvegicus* | 124.7166972 | 37.82273889 |
| 274 | *Rattus norvegicus* | 125.7033111 | 37.66264167 |
| 275 | *Rattus norvegicus* | 127.1358389 | 37.68009444 |
| 276 | *Rattus norvegicus* | 127.3089889 | 37.56003611 |
| 277 | *Rattus norvegicus* | 127.1297806 | 37.5856 |
| 278 | *Rattus norvegicus* | 35.51136389 | 127.7828581 |
| 279 | *Rattus norvegicus* | 35.51685306 | 127.8975219 |
| 280 | *Rattus norvegicus* | 35.51446 | 128.71341 |
| 281 | *Rattus norvegicus* | 35.53748306 | 127.8144611 |
| 282 | *Rattus norvegicus* | 35.53409 | 128.7023 |
| 283 | *Rattus norvegicus* | 35.56513611 | 127.9834889 |
| 284 | *Rattus norvegicus* | 35.55944389 | 128.8555561 |
| 285 | *Rattus norvegicus* | 35.56555611 | 128.8863889 |
| 286 | *Rattus norvegicus* | 35.59164194 | 127.8549111 |
| 287 | *Rattus norvegicus* | 35.59652194 | 127.9769061 |
| 288 | *Rattus norvegicus* | 35.63290306 | 127.8630919 |
| 289 | *Rattus norvegicus* | 35.62787 | 128.76377 |
| 290 | *Rattus norvegicus* | 35.67413 | 128.86829 |
| 291 | *Rattus norvegicus* | 35.71085611 | 127.6911081 |
| 292 | *Rattus norvegicus* | 35.73784694 | 127.8555361 |
| 293 | *Rattus norvegicus* | 35.72672 | 128.85027 |
| 294 | *Rattus norvegicus* | 36.39111111 | 126.3380561 |
| 295 | *Rattus norvegicus* | 36.42305611 | 126.4086111 |
| 296 | *Rattus norvegicus* | 36.4475 | 126.3366669 |
| 297 | *Rattus norvegicus* | 36.45555611 | 126.4972219 |
| 298 | *Rattus norvegicus* | 36.4675 | 126.5783331 |
| 299 | *Rattus norvegicus* | 36.48222194 | 126.5275 |
| 300 | *Rattus norvegicus* | 36.48388889 | 126.5980561 |
| 301 | *Rattus norvegicus* | 36.4925 | 126.3388889 |
| 302 | *Rattus norvegicus* | 36.54861111 | 126.4716669 |
| 303 | *Rattus norvegicus* | 36.60888889 | 126.4208331 |
| 304 | *Rattus norvegicus* | 36.703065 | 126.5704081 |
| 305 | *Rattus norvegicus* | 36.76083306 | 126.9180831 |
| 306 | *Rattus norvegicus* | 36.76377806 | 126.8957219 |
| 307 | *Rattus norvegicus* | 36.76644389 | 127.1067781 |
| 308 | *Rattus norvegicus* | 36.78191694 | 126.9186111 |
| 309 | *Rattus norvegicus* | 36.78922194 | 127.0227781 |
| 310 | *Rattus norvegicus* | 36.80052806 | 126.9214169 |
| 311 | *Rattus norvegicus* | 36.80230611 | 127.0476939 |
| 312 | *Rattus norvegicus* | 36.80194611 | 126.4922011 |
| 313 | *Rattus norvegicus* | 36.80841694 | 127.0816389 |
| 314 | *Rattus norvegicus* | 36.81055611 | 127.0958611 |
| 315 | *Rattus norvegicus* | 36.81566694 | 126.99125 |
| 316 | *Rattus norvegicus* | 36.81672194 | 126.8995281 |
| 317 | *Rattus norvegicus* | 36.82011111 | 127.0201389 |
| 318 | *Rattus norvegicus* | 36.85147194 | 127.0785831 |
| 319 | *Rattus norvegicus* | 36.86405611 | 127.0955281 |
| 320 | *Rattus norvegicus* | 36.87133306 | 127.0040561 |
| 321 | *Rattus norvegicus* | 36.87936111 | 127.0994719 |
| 322 | *Rattus norvegicus* | 36.88469389 | 127.0048889 |
| 323 | *Rattus norvegicus* | 36.88525611 | 126.6437611 |
| 324 | *Rattus norvegicus* | 36.88713889 | 127.0537781 |
| 325 | *Rattus norvegicus* | 36.89111694 | 126.9781419 |
| 326 | *Rattus norvegicus* | 36.89438889 | 126.6850969 |
| 327 | *Rattus norvegicus* | 36.90486111 | 127.0087781 |
| 328 | *Rattus norvegicus* | 36.91452806 | 127.0430281 |
| 329 | *Rattus norvegicus* | 36.93369194 | 126.9439831 |
| 330 | *Rattus norvegicus* | 36.94002194 | 126.7423439 |
| 331 | *Rattus norvegicus* | 36.94719389 | 127.0268889 |
| 332 | *Rattus norvegicus* | 36.93294389 | 129.2334719 |
| 333 | *Rattus norvegicus* | 36.95705611 | 127.1054439 |
| 334 | *Rattus norvegicus* | 36.96016694 | 127.09725 |
| 335 | *Rattus norvegicus* | 36.97372194 | 127.0273061 |
| 336 | *Rattus norvegicus* | 36.98461111 | 127.0872219 |
| 337 | *Rattus norvegicus* | 36.98908306 | 127.0049719 |
| 338 | *Rattus norvegicus* | 37.58406806 | 126.5942361 |
| 339 | *Rattus norvegicus* | 37.75447194 | 126.5321189 |
| 340 | *Rattus norvegicus* | 37.78816111 | 126.3658081 |
| 341 | *Rattus norvegicus* | 37.83818 | 124.701605 |
| 342 | *Rattus norvegicus* | 37.84220306 | 124.70727 |
| 343 | *Rattus norvegicus* | 34.886222 | 127.290361 |
| 344 | *Rattus norvegicus* | 34.779444 | 127.27525 |
| 345 | *Rattus norvegicus* | 34.810139 | 127.357472 |
| 346 | *Rattus norvegicus* | 34.868361 | 127.017889 |
| 347 | *Rattus norvegicus* | 37.51311111 | 130.9091944 |
| 348 | *Rattus norvegicus* | 35.51730556 | 128.9324444 |
| 349 | *Rattus norvegicus* | 35.58755556 | 128.9961389 |
| 350 | *Rattus norvegicus* | 35.705 | 129.0156944 |
| 351 | *Rattus norvegicus* | 35.69552778 | 129.07325 |
| 352 | *Rattus norvegicus* | 35.10137336 | 126.6083664 |
| 353 | *Rattus norvegicus* | 34.73410889 | 126.8153322 |
| 354 | *Rattus norvegicus* | 34.81052234 | 126.780108 |
| 355 | *Rattus norvegicus* | 35.12674148 | 126.624259 |
| 356 | *Rattus norvegicus* | 37.64888889 | 128.1413889 |
| 357 | *Rattus norvegicus* | 37.64908333 | 128.143 |
| 358 | *Rattus norvegicus* | 37.92508719 | 127.638498 |
| 359 | *Rattus norvegicus* | 34.610949 | 126.688854 |
| 360 | *Rattus norvegicus* | 34.52777 | 126.736068 |
| 361 | *Rattus norvegicus* | 34.615126 | 126.758598 |
| 362 | *Rattus norvegicus* | 34.607476 | 126.733802 |
| 363 | *Rattus norvegicus* | 34.472577 | 126.2655 |
| 364 | *Rattus norvegicus* | 34.437925 | 126.283708 |
| 365 | *Rattus norvegicus* | 34.471827 | 126.359224 |
| 366 | *Rattus norvegicus* | 35.72408333 | 128.7414722 |
| 367 | *Rattus norvegicus* | 35.14184444 | 128.0587222 |
| 368 | *Rattus norvegicus* | 36.31908333 | 128.9840556 |
| 369 | *Rattus norvegicus* | 36.32005556 | 128.9800833 |
| 370 | *Rattus norvegicus* | 35.27026322 | 128.4975728 |
| 371 | *Rattus norvegicus* | 35.27180159 | 128.4550001 |
| 372 | *Rattus norvegicus* | 37.97581589 | 127.4410993 |
| 373 | *Rattus norvegicus* | 37.17366111 | 127.1815333 |
| 374 | *Rattus norvegicus* | 37.05807222 | 127.3633306 |
| 375 | *Rattus norvegicus* | 37.03423056 | 127.1771222 |
| 376 | *Rattus norvegicus* | 34.636536 | 126.812742 |
| 377 | *Rattus norvegicus* | 33.2402 | 126.5589194 |
| 378 | *Rattus norvegicus* | 34.88369444 | 128.4928889 |
| 379 | *Rattus norvegicus* | 33.50827778 | 126.4699444 |
| 380 | *Rattus norvegicus* | 33.50038889 | 126.90525 |
| 381 | *Rattus norvegicus* | 33.50847222 | 126.5987222 |
| 382 | *Rattus norvegicus* | 33.45621111 | 126.710075 |
| 383 | *Rattus norvegicus* | 33.45621944 | 126.7100639 |
| 384 | *Rattus norvegicus* | 33.45618306 | 126.7101278 |
| 385 | *Rattus norvegicus* | 33.45624417 | 126.7101444 |
| 386 | *Rattus norvegicus* | 37.54628889 | 126.3305778 |
| 387 | *Rattus norvegicus* | 37.53120556 | 126.3360278 |
| 388 | *Rattus norvegicus* | 37.53130556 | 126.372975 |
| 389 | *Rattus norvegicus* | 37.27805556 | 126.4855083 |
| 390 | *Rattus norvegicus* | 37.23258056 | 126.51625 |
| 391 | *Rattus norvegicus* | 37.20989444 | 126.5609694 |
| 392 | *Rattus norvegicus* | 37.22334167 | 126.5891 |
| 393 | *Rattus norvegicus* | 37.17148611 | 126.6236694 |
| 394 | *Rattus norvegicus* | 37.40591111 | 126.7762833 |
| 395 | *Rattus norvegicus* | 37.40995278 | 126.8455722 |
| 396 | *Rattus norvegicus* | 37.45541389 | 126.8202389 |
| 397 | *Rattus norvegicus* | 37.45995278 | 126.8536444 |
| 398 | *Rattus norvegicus* | 37.45115833 | 127.02945 |
| 399 | *Rattus norvegicus* | 37.40432222 | 127.0105028 |
| 400 | *Rattus norvegicus* | 36.06499405 | 126.6838391 |
| 401 | *Rattus norvegicus* | 35.92446714 | 126.7657371 |
| 402 | *Rattus norvegicus* | 35.88736436 | 126.727265 |
| 403 | *Rattus norvegicus* | 35.80047369 | 126.4284366 |
| 404 | *Rattus norvegicus* | 35.10163056 | 128.9387306 |
| 405 | *Rattus norvegicus* | 35.24099444 | 128.7046028 |
| 406 | *Rattus norvegicus* | 35.18969444 | 128.7231944 |
| 407 | *Rattus norvegicus* | 35.16291944 | 128.6793833 |
| 408 | *Rattus norvegicus* | 35.14803333 | 128.6763361 |
| 409 | *Rattus norvegicus* | 35.12029444 | 128.7454361 |
| 410 | *Rattus norvegicus* | 35.01678056 | 128.7032333 |
| 411 | *Rattus norvegicus* | 35.02072222 | 128.7195833 |
| 412 | *Rattus norvegicus* | 36.11941389 | 128.860225 |
| 413 | *Rattus norvegicus* | 36.07272778 | 128.8397944 |
| 414 | *Rattus norvegicus* | 36.14311667 | 128.5392361 |
| 415 | *Rattus norvegicus* | 34.466851 | 127.224104 |

TABLE S2. Environmental variables used for habitat suitability modelling of brown rat (BR) in South Korea.

| **Variable Category** | **Code** | **Variable** | **Unit** | **Source** |
| --- | --- | --- | --- | --- |
| **Bio-Climatic** | Bio01 | Annual mean temperature | ˚C | https://www.worldclim.org/ |
|  | Bio02 | Mean diurnal range  (Mean of monthly (max temp -min temp)) | ˚C | https://www.worldclim.org/ |
|  | Bio03 | Isothermality (Bio02/Bio07) (x100) | - | https://www.worldclim.org/ |
|  | Bio04 | Temperature seasonality  (standard deviation x 100) | - | https://www.worldclim.org/ |
|  | Bio05 | Max temperature of warmest Month | ˚C | https://www.worldclim.org/ |
|  | Bio06 | Min temperature of coldest month | ˚C | https://www.worldclim.org/ |
|  | Bio07 | Temperature annual range (Bio05 - Bio06) | ˚C | https://www.worldclim.org/ |
|  | Bio08 | Mean temperature of wettest quarter | ˚C | https://www.worldclim.org/ |
|  | Bio09 | Mean temperature of driest quarter | ˚C | https://www.worldclim.org/ |
|  | Bio10 | Mean temperature of warmest quarter | ˚C | https://www.worldclim.org/ |
|  | Bio11 | Mean temperature of coldest quarter | ˚C | https://www.worldclim.org/ |
|  | Bio12 | Annual precipitation | mm | https://www.worldclim.org/ |
|  | Bio13 | Precipitation of wettest month | mm | https://www.worldclim.org/ |
|  | Bio14 | Precipitation of driest month | mm | https://www.worldclim.org/ |
|  | Bio15 | Precipitation seasonality (Coefficient of variation) | - | https://www.worldclim.org/ |
|  | Bio16 | Precipitation of wettest quarter | mm | https://www.worldclim.org/ |
|  | Bio17 | Precipitation of driest quarter | mm | https://www.worldclim.org/ |
|  | Bio18 | Precipitation of warmest quarter | mm | https://www.worldclim.org/ |
|  | Bio19 | Precipitation of coldest quarter | mm | https://www.worldclim.org/ |
| **Topographic** | Elev | Elevation | m | https://www.worldclim.org/ |
|  | Slope | Slope | ˚ | - |
|  | Aspect | Aspect | ˚ | - |
|  | East | East-ness (as a measure of aspect) | - | - |
|  | North | North-ness (as measure of aspect) | - | - |
| **Habitat- related** | LC | Land cover | - | - |
|  | Dist_barren | Distance from barren land | m | - |
|  | Dist_crop | Distance from crop | m | - |
|  | Dist_grassland | Distance from grassland | m | - |
|  | Dist_forest | Distance from forest | % | - |
|  | Dist_water | Distance from water | m | - |
|  | Prop_crop | Proportion to Crop | % | - |
|  | Prop_deci | Proportion of deciduous forest | % | - |
|  | NPP | Net primary production | gCm^-2^yr^-1^ | https://ladweb.modaps.eosdis.nasa,gov/ |
|  | NDVI | Annual mean normalized difference vegetation index | - | https://modis.gsfc.nasa.gov/ |
|  | Nightlight | Nighttime light | - | htpps://www.resdc.cn/ |
|  | Soil coarse fragmentation | Coarse fragmentation volumetric | %vol | https://zenodo.org/ |
|  | Footprint | Global human footprint | - | https://sedac.ciesin.columbia.edu/ |
|  | LULC | Future land use and Land cover | - | https://www.resdc.cn/ |


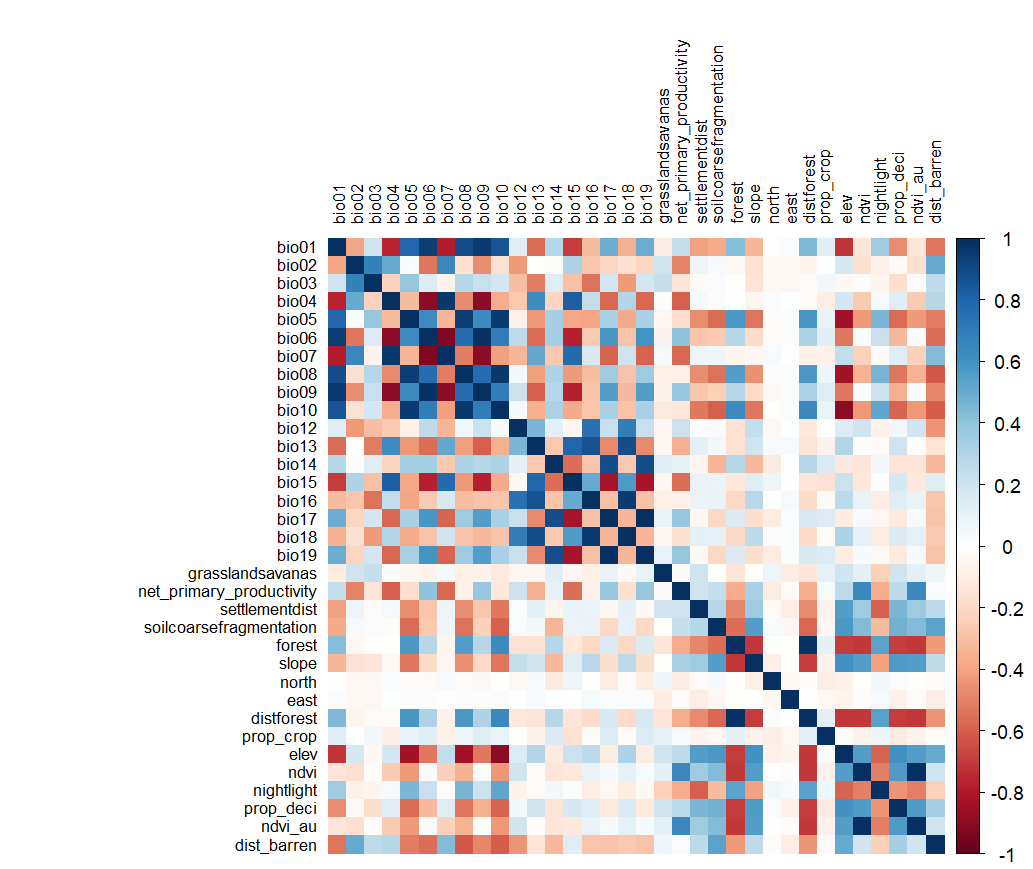


FIGURE S2 Correlation matrix plot of selected variables for habitat suitability modelling of Brown rat (BR). Highly correlated variables (r ≥ |0.75|) were excluded for the final distribution model.


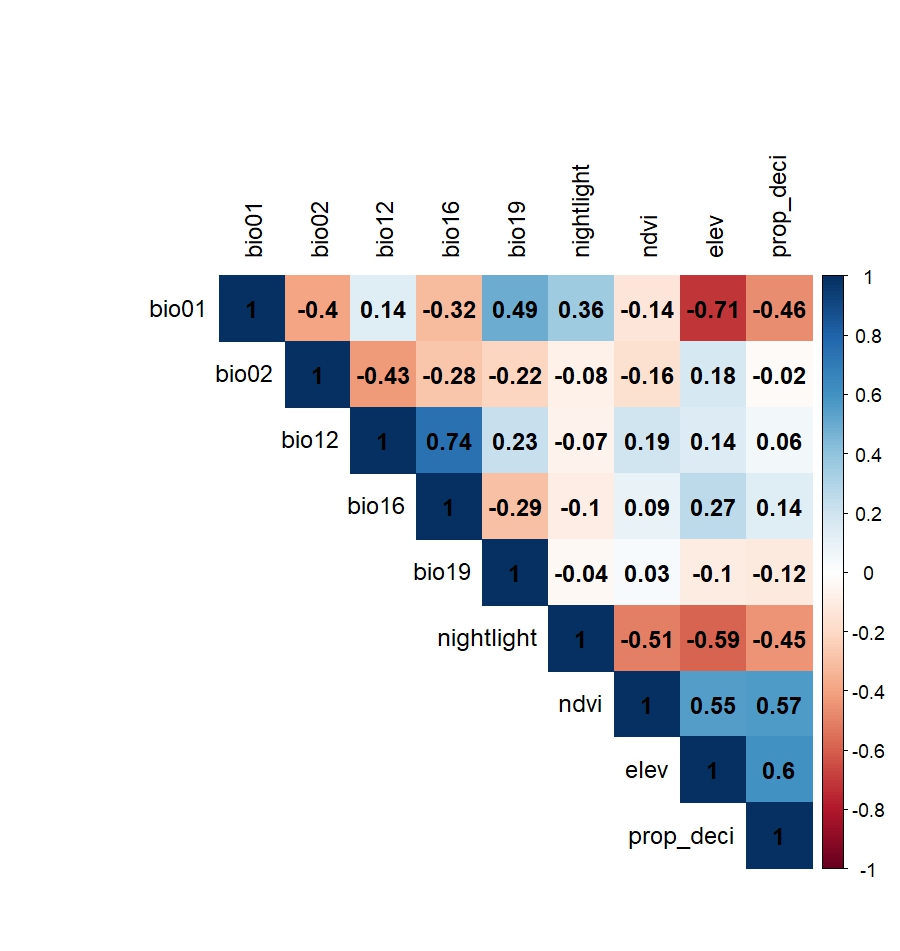


FIGURE S3 Spearman’s Rank Correlation between the final set of variables used for the ensemble models for the brown rat (BR). The variables are coded as bio01: Annual mean temperature, Bio02: Mean diurnal range, Bio12: Annual precipitation, Bio16: Precipitation of wettest quarter, Bio19: Precipitation of coldest quarter, nightlight: Nighttime light, elev: Elevation, ndvi: Normalized difference vegetation index, and prop_deci: Proportion of deciduous forest.

TABLE S3. Distribution changes of brown rat (BR) based on binary distribution for 2030s and 2050s under the shared socioeconomic pathways (SSPs) 1-2.6 and 5-8.5 including dispersal scenarios.

| **Year** | **Climatic Scenario** | **Dispersal**  **Scenario** | | **Habitat**  **Area (km^2^)** | **Expansion**  **(km^2^)** | **Unchanged**  **(km^2^)** | **Contraction**  **(km^2^)** |
| --- | --- | --- | --- | --- | --- | --- | --- |
| **2030s** | **SSP 1-2.6** | Unlimited | | 36460 | 6396 | 30055 | 11030 |
|  |  | Limited | 1km | 28932 | 5201 | 45825 | 7390 |
|  |  |  | 5km | 28932 | 5516 | 45710 | 7390 |
|  |  |  | 10km | 28932 | 5535 | 45704 | 7390 |
|  | **SSP 5-8.5** | Unlimited | | 37581 | 23308 | 35313 | 2264 |
|  |  | Limited | 1km | 38809 | 21684 | 27036 | 1157 |
|  |  |  | 5km | 38809 | 23121 | 27035 | 1157 |
|  |  |  | 10km | 38809 | 23167 | 27035 | 1157 |
| **2050s** | **SSP 1-2.6** | Unlimited | | 44898 | 9453 | 32756 | 12132 |
|  |  | Limited | 1km | 28932 | 8933 | 45825 | 1840 |
|  |  |  | 5km | 28932 | 9992 | 45710 | 1954 |
|  |  |  | 10km | 28932 | 10007 | 45705 | 1960 |
|  | **SSP 5-8.5** | Unlimited | | 48473 | 31622 | 39996 | 1090 |
|  |  | Limited | 1km | 38809 | 10035 | 27035 | 27 |
|  |  |  | 5km | 38809 | 9477 | 27035 | 28 |
|  |  |  | 10km | 38809 | 9519 | 27035 | 28 |

TABLE S4. centroid coordinates and shift range of brown rat (BR*)* in the South Korea.

| **Climate Scenario** | **Period** | **Location** | **Longitude** | **Latitude** | **Range Shift (km)** | **Shift direction (˚)** | **Shifting rate (km/year)** |
| --- | --- | --- | --- | --- | --- | --- | --- |
|  | Current | Jeoksang-myeon | 127° 35' 38.4" | 35° 57' 36" | - | - | - |
| **SSP 1-2.6** | 2030s | Cheoncheon-myeon | 127° 32' 21" | 35° 46' 2" | 22.40 | 196.2 | 1.12 |
|  | 2050s | Yongdam-myeon | 127° 29' 6" | 35° 55' 59" | 18.97 | 342.1 | 0.95 |
| **SSP 5-8.5** | 2030s | Jewon-myeon | 12° 35' 13.2" | 35° 6' 7" | 15.71 | 356.8 | 0.75 |
|  | 2050s | Yangsan-myeon | 127° 37' 55.2" | 36° 9' 43" | 7.89 | 36.7 | 0.39 |
